# Barriers to Early Detection and Management of Gestational Diabetes Mellitus in Northwest Ethiopia: A Qualitative Study From the Healthcare Providers’ Perspectives

**DOI:** 10.1155/ogi/1739878

**Published:** 2026-08-27

**Authors:** Getachew Tilaye Mihiret, Kumlachew Solomon Wondmu, Fekadu Baye, Mulunesh Minale, Mastewal Yechale, Misganaw Fikirie Melese, Yilkal Dagnaw Melesse, Aysheshim Belaineh Haimanot, Aderajew Agmass Adebabay, Haile Amha

**Affiliations:** ^1^ Department of Midwifery, College of Medicine and Health Sciences, Debre Markos University, P.O. Box 269, Debre-Markos, Ethiopia, dmu.edu.et; ^2^ Department of Public Health, College of Medicine and Health Sciences, Debre Markos University, P.O. Box 269, Debre-Markos, Ethiopia, dmu.edu.et; ^3^ Department of Biomedical Sciences, College of Medicine and Health Sciences, Debre Markos University, P.O. Box 269, Debre-Markos, Ethiopia, dmu.edu.et; ^4^ Department Nursing, College of Medicine and Health Sciences, Debre Markos University, Debre Markos, Ethiopia, dmu.edu.et

**Keywords:** barriers, Ethiopia, gestational diabetes, gestational diabetes mellitus, management, screening

## Abstract

**Background:**

Gestational diabetes mellitus (GDM) is a major public health challenge associated with adverse maternal and neonatal outcomes. In low‐income countries such as Ethiopia, screening rates remain critically low, resulting in preventable complications and long‐term burdens on the health system.

**Aims:**

This qualitative study examines healthcare providers’ perspectives on barriers to the early diagnosis and management of GDM in northwest Ethiopia.

**Methods:**

A descriptive qualitative study was conducted among eight healthcare providers (midwives, an obstetrician, and general practitioners) delivering maternal health services at public hospitals in the Eastern Gojjam zone. Participants were purposively selected, and data were collected through in‐depth interviews. Interviews were audio‐recorded, transcribed, translated, and analyzed using inductive thematic analysis in NVivo 12.2. The socioecological model guided the synthesis of barriers across multiple levels.

**Results:**

Providers reported a cascade of barriers spanning individual, community, and health system levels. Health system level barriers were most prominent, including shortages of trained staff, lack of essential supplies (glucometers, test strips), overcrowding leading to prolonged waiting times, and the absence of preconception care services. At the individual level, late antenatal booking and limited patient awareness hindered timely diagnosis. Community‐level barriers included cultural beliefs that fostered denial of diagnosis and reliance on traditional remedies, further impeding care engagement.

**Conclusion and Recommendations:**

Early detection and management of GDM in Northwest Ethiopia remain constrained by a complex interplay of individual knowledge gaps, cultural norms, and systemic health service weaknesses. Addressing these challenges requires a comprehensive health systems approach that concurrently strengthens infrastructure, expands human resource capacity, and fosters community engagement. Such an integrated strategy is essential to translate national policy into equitable and effective practice, ultimately improving maternal and neonatal outcomes.

## 1. Introduction

Pregnancy is characterized by physiological insulin resistance, which may expose gestational diabetes mellitus (GDM) [1]. Several factors are contributing to hyperglycemia during pregnancy, including placental hormones such as human placental lactogen, and the insulin‐antagonistic effects of progesterone and estrogen. Increased maternal adipose tissue deposition, reduced physical activity, and higher caloric intake are also additional contributing factors [2, 3].

A recent systematic review reported that the burden of GDM in sub‐Saharan Africa is approximately 14.3%, underscoring its growing public health significance in the region. Similarly, a study conducted in Northwest Ethiopia found that nearly 13% of pregnant women were diagnosed with GDM [4]. These findings highlight that GDM represents an under‐recognized challenge to maternal and neonatal health in low‐ and middle‐income countries (LMICs), including Ethiopia, where health systems often face resource constraints and limited screening capacity.

Hyperglycemia during pregnancy is associated with substantial adverse outcomes for both mothers and their offspring. Maternal complications include an increased risk of cesarean delivery, pregnancy‐induced hypertension, premature rupture of membranes, antepartum hemorrhage, and postpartum hemorrhage [5–8]. Infants born to mothers with hyperglycemia are at greater risk of macrosomia, respiratory distress syndrome, preterm birth, neonatal jaundice, hypoglycemia, stillbirth, and admission to the neonatal intensive care unit [7, 9–11]. Beyond these immediate consequences, intrauterine exposure to GDM has been linked to an increased lifetime risk of obesity, hypertension, and type 2 diabetes among offspring [6, 12], underscoring the intergenerational impact of maternal hyperglycemia.

International guidelines, such as those from the World Health Organization (WHO) and International Federation of Gynecology and Obstetrics (FIGO), recommend universal screening via a 75‐g oral glucose tolerance test [13, 14]. While this is standard practice in high‐income countries, a profound implementation gap exists in LMICs like Ethiopia, which bear a disproportionate burden of undiagnosed GDM and its adverse outcomes [15–17].

GDM screening is usually done between 24 and 28 weeks of pregnancy [18], because different placental hormones promote insulin resistance during pregnancy [2]. However, screening may be performed earlier in pregnancy if there are risk factors such as a history of GDM, obesity, urinary glucose excretion, and a strong family history of diabetes [19].

Timely screening and effective management of GDM are critical public health interventions. However, a variety of challenges hinder the early detection and treatment of hyperglycemia during pregnancy, particularly in LMICs [20, 21]. Research from African settings identifies this gap not merely as a clinical oversight but as a consequence of multifaceted health system barriers. These include resource constraints (lack of glucometers, test strips, trained staff), organizational challenges (overcrowding, long wait times, absent protocols), and socioeconomic factors (drug cost, transportation) [22–24].

Awareness gaps among both patients and healthcare providers are also a major barrier to the early detection and management of GDM. Research from Ethiopia reported that only 27% of pregnant women are aware of diabetes during pregnancy [25]. In Ethiopia, despite the rising burden of GDM, the quality and operational realities of screening and management services are poorly documented and understood [20, 21]. Crucially, there is a lack of in‐depth insight into the frontline healthcare providers’ perspectives those who navigate these systemic barriers daily. Understanding their experiences is essential for designing feasible, context‐appropriate interventions.

Therefore, this study aimed to explore the perspectives of healthcare providers on the barriers to early detection and management of GDM in Northwest Ethiopia. Guided by the socioecological model, we sought to systematically identify challenges at the individual, organizational, community, and policy levels. The findings are intended to generate evidence‐based, multilevel management recommendations to inform the development of sustainable screening and management programs within the Ethiopian health system, thereby improving diabetes outcomes.

## 2. Methods

*Reporting protocols*: The reporting of this qualitative study followed the Consolidated Criteria for Reporting Qualitative Research (COREQ) checklist, a 32‐item framework encompassing three domains: research team and reflexivity, study design, and data analysis and reporting. Application of the COREQ framework ensured transparent and comprehensive reporting of the research context, data collection procedures, analytical processes, and interpretation of findings [26].

### 2.1. Research Team and Reflexivity

Data were collected by four researchers with prior experience in qualitative research methods and familiarity with the study topic. All researchers were proficient in both Amharic and English and held a master’s degree in maternity and reproductive health nursing. None of the researchers had a prior relationship or interaction with the study participants before data collection commenced.

### 2.2. Data Quality and Trustworthiness

To ensure the credibility and dependability of the study, different key strategies were employed. Interviewers were trained on the study overview and interview approach. The four criteria, such as credibility, dependability, confirmability, and transferability, were applied to ensure the trustworthiness of the data [27].

Credibility of the data was assured by checking the interview guide for appropriateness of wording before the data collection. Rapport was building via persistent observation and prolonged engagement with participants, and interviewers took ample time to probe respondents for deeper responses, allowing them to express themselves more freely. During the interviews, the sound was recorded, and notes were taken, with consideration of nonverbal communication (the feelings of participants).

Dependability of the data was assured by using the code–recode procedure, employing a similar semi‐structured interview guide, and utilizing two trained data collectors at a time. Supervision was conducted throughout the data collection process by the lead researcher.

To assure the confirmability, transcriptions of the data were completed on the day of data collection, and responses were cross‐checked to ensure that the results accurately echoed the respondents’ viewpoints. Transferability of the findings was established through a detailed description of methodology, including the sampling technique, data collection procedures, the process of data analysis, and the study findings, thus allowing the researcher affirm the applicability of the findings to their own contexts. The findings are also supported with meaningful quotes.

### 2.3. Study Design and Period

This study employed an exploratory qualitative research design to explore barriers to early detection and management of GDM among healthcare providers. This approach involves trying to understand the essence of the issue by examining the experience of healthcare providers and leadership working in maternal and child care.

### 2.4. Study Area and Period

The study was carried out over a 3‐month period, between December 2024 and February 2025, in public hospitals in East Gojjam. The area is located in Ethiopia’s Amhara region, which is bounded to the south by Oromia, to the west‐by‐West Gojjam, to the north by Debub‐Gondar, and to the east by Debub Wollo. The area’s population is estimated to be 2,719,118 inhabitants in 2020 [28]. In this area, the population is predominantly Orthodox Christian. The zone has 10 public hospitals, including 9 primary hospitals, and 1 specialized hospital, as well as 102 health centers and 406 health posts [29].

### 2.5. Participants

The study participants comprised health professionals working in maternal and child health settings, such as midwives, general practitioners, and obstetricians, as well as healthcare providers working in the area of healthcare leadership who had 1 year or more of working experience. A purposive sampling technique was employed to select participants. The participants had about 8 years’ average working experience, with some having up to 12 years in the field.

### 2.6. Sample Size and Sampling Technique

The sample size was determined using the principle of data saturation, whereby participant recruitment continued until no new information or themes emerged and data redundancy was achieved [30]. Based on this approach, eight participants were included in the in‐depth interviews. Participants were selected purposively based on their experience and direct involvement in GDM screening and management as primary caregivers.

Three public hospitals Dejen Primary Hospital, Motta General Hospital, and Debre Markos Comprehensive Specialized Hospital were purposively selected from the 11 public hospitals in East Gojjam Zone to capture diverse service delivery contexts. Potential participants were initially contacted by telephone, followed by face‐to‐face communication to provide detailed information about the study. Eligible participants were then invited by the research team to take part in interviews at a mutually convenient time and location.

Data saturation was assessed iteratively during data collection and preliminary analysis. Recruitment was discontinued when successive interviews yielded no new themes and thematic redundancy was confirmed.

### 2.7. Data Collection Procedures

The interview guide was developed based on a thorough review of the literature, complemented by expert input. To ensure clarity, relevance, and cultural appropriateness, the guide was pilot‐tested with two individuals from a similar demographic. Based on their feedback, minor revisions were made to improve question flow and reduce ambiguity. Trained researchers conducted the data collection using a semi‐structured interview guide and audio recording. The interview guide was prepared in the English version and then translated into the Amharic version (local language). The interview was done in Amharic language, and on average, the interview lasted for around 30–45 min.

In‐depth interviews were employed as the data collection method to capture the barriers for detection and management of GDM. This method elicits candid responses in a private setting regarding professional topics of discussion. It is useful to have each participant have more time and opportunity to share feelings, perspectives, and experiences concerning the problem. Also, an in‐depth interview is appropriate for this study since we are interviewing healthcare providers with specific knowledge and experience concerning the issue, and it is better suited to sitting down one‐on‐one. It also allows considerable opportunity to probe answers and for intensive investigation of individual experiences and thoughts.

The interview guide was created in various stages by employing the socioecological model, covering topics on perceived importance of GDM screening, methods of screening, current practice of screening, knowledge of healthcare providers and women about GDM, screening process of GDM, the role of healthcare providers in GDM managements, and the available resources (Supporting File. 1).

### 2.8. Data Management

Immediately following each interview, the recordings were listened to several times, and the interview was transcribed verbatim into Amharic (the local language). The principal investigator translated the transcripts into English, and the transcriptions and translations were spot‐checked, and the analysis was performed using the English transcript.

Grounded theory was used to analyze the data. In grounded theory, coding is divided into iterative stages. The coding terminology in evolved grounded theory includes open coding for developing categories, axial coding for connecting those categories, and selective coding for creating a narrative that links the core categories together, leading to a discursive collection of theoretical propositions, ultimately resulting in a set of theoretical propositions [31].

This study utilized evolved genres to outline each coding phase; accordingly, to create a cohesive narrative from the empirical data, we coded transcripts, labeled and categorized concepts, linked categories and subcategories, and combined the primary categories. Throughout the coding, the participant’s quotation was used to spot‐check the identified categories, ensuring that the finding is firmly based on the interview data.

The socioecological model was employed to connect themes and to understand the interplay between personal, household, cultural, and health system barriers that inhibit timely screening and management of GDM. For analysis, the data were imported into Nvivo version 12.2 software packages. The software was used to code the data in each category depending on their differences and similarities.

### 2.9. Ethical Consideration

Ethical clearance was found from the Institutional Review Board of Debre Markos University, College of Medicine and Health Sciences (Approval No.: CMHS/R/C/Ser/D/315/01/16), in accordance with the Declaration of Helsinki and applicable national guidelines. Responsible officials and managers at each hospital were communicated, and permission was obtained. All participants interviewed provided informed written consent to participate in the interviews, permission to record interviews, and publish anonymized data in journals, reports, etc. The participant’s detailed information and the transcripts were kept in password‐protected file. No financial or material compensation was provided to participants. Their involvement was entirely voluntary, and they were informed of their right to withdraw at any stage without consequence.

## 3. Result

### 3.1. Characteristics of Participants

A total of eight healthcare professionals were interviewed for this qualitative study, including two obstetricians and gynecologists, one general practitioner, and five midwives aged 29–42 years. All belonged to the orthodox Christian religion. Each participant satisfied our selection requirements. After interviewing eight healthcare providers, we came to an end to recruitment since they satisfied our study requirements and we achieved saturation with regard to the depth of data we were trying to uncover (Table 1).

**TABLE 1:** Sociodemographic and professional characteristics of study participants in East Gojjam zone public hospitals, Northwest Ethiopia, 2025.

No.	Sex	Age	Designation	Education	Experience
IDI002	F	30	Senior midwife	MSc in clinical midwifery	6 years
IDI003	M	29	Senior midwife	BSc midwife	7 years
IDI001	F	32	Senior midwife	BSc midwife	8 years
IDI005	F	35	Senior midwife	Senior midwife	8 years
IDI004	M	34	Senior midwife	Diploma midwife	6 years
IDI006	M	42	Obstetrician	Obstetrician specialist	11 years
IDI007	M	38	General practitioner	General practitioner	7 years
IDI008	M	45	Obstetrician	Obstetrician specialist	12 years

### 3.2. Perceived Importance of GDM Screening

The importance of early screening and diagnosis of GDM in pregnant women was stressed by all study participants. They stressed the importance of early detection of GDM in order to manage preventable complications during pregnancy and childbirth. One attendee mentioned the following:

It’s useful for many things. It’s good for both the mother and the child. It’s useful in managing the fetus to know what to do, it can be the delivery system, it can be the diet, it can help us decide what to do. It is also useful for providing individualized (client‐centered) advice (IDI#2, Clinical midwife, age 30).

Another participant explained the main benefits of detecting the GDM status of a woman by screening as follows:

What question does have? (Screening of GDM); For example, a mother with GDM is more likely to have a high birth weight newborn, which can lead to various complications. Therefore, screening of pregnant women for GDM is useful for early treatment of the disease. This protects the child and the family from various expenses (IDI#5, senior midwife, age 35).

GDM can affect both the mother and baby, increasing the risk of complications during pregnancy and delivery, as well as potential long‐term health issues for the baby. Study participants noted the benefit of early diagnosis of GDM in order to manage concomitant complications of diabetes mellitus during pregnancy and childbirth. One respondent mentioned the following:

“Gestational diabetes mellitus affects women not only during pregnancy but also after pregnancy. Gestational diabetes affects all organs of the mother, primarily the renal system, eyes, and nervous system. In addition, gestational diabetes may persist after delivery and progress to type‐2 diabetes. It may also weaken the immune system, leading to various infections. The most common effect on the fetus is congenital anomalies; all organs can be affected, although it is more prevalent in the cardiovascular and renal systems. The fetus may become macrosomia, which can lead to shoulder dystocia, perineal tears, and cervical tears” (IDI#6, general practitioner, age 38).

### 3.3. Detection of GDM

All participating professionals were asked about the screening procedures for GDM in their respective hospitals. All participants except those from Debre Markos comprehensive specialized hospital use a selective screening method to determine blood sugar levels in pregnant women if they show signs and symptoms of diabetes during pregnancy. One of the participants commented on the selective screening method as follows:

“We don’t routinely screen all pregnant women at our hospital, but if a mother shows signs and symptoms, we screen the mother for GDM by using a random blood sugar level” (IDI#4, senior midwife, age 34).

Another participant also added his comment:

“We do not routinely screen pregnant women for GDM, it is a rare case, we only screen them when they show signs of GDM” (IDI#2, senior midwife, age 29).

All participants from Debre Markos comprehensive specialized hospital echoed the benefit of early screening of GDM, and they use universal screening of all women when they come for ANC visit, at 24–28 weeks of pregnancy, and one participant said:

“We use universal screening at our hospital, irrespective of their risk assessment. At 26 weeks of pregnancy, we sent a random blood sample to laboratory for further testing. We know that GDM has undesirable perinatal consequences if not identified early. We screen all pregnant women routinely by using random blood sample (RBS) (IDI#6, general practitioner, age 38).”

### 3.4. Current Screening Practices

Several organizations, including the WHO and the FIGO, have established guidelines for the screening and management of GDM. Both WHO and FIGO advocate universal screening of GDM for all pregnant women to be screened from 24 to 28 weeks of gestation regardless of risk factors [14], while Ethiopia’s guidelines recommend a selective screening for GDM if women are at high risk for GDM [17]. The WHO and FIGO agree on using the OGTT as the gold standard for diagnosis; Ethiopia’s GDM screening and management guideline also take on this recommendation [13].

All participants from primary and general hospitals reported that national screening guidelines are not routinely implemented. Consequently, providers do not practice the recommended single‐step screening for GDM using a 75‐g oral glucose solution. Instead, women are screened only when presenting with signs or symptoms of hyperglycemia, typically through random blood glucose testing. To contextualize these practices, Table 2 presents a comparative analysis of the Ethiopian Ministry of Health’s (MoH) GDM screening and management protocol alongside international recommendations from WHO and FIGO. The comparison focuses on screening methods, diagnostic criteria, management strategies, and follow‐up care, highlighting areas of alignment and divergence across the three protocols.

**TABLE 2:** Comparative analysis of GDM screening and diagnostic criteria: Ethiopian National Guidelines vs. WHO and FIGO standards.

Guidelines	Screening/diagnosis	Management
Ethiopia’s guideline	Screening: If a woman has one or more of risk factors for GDM, perform a one‐step screening using 75 g of 2‐h OGTT as soon as feasible. If GDM is not diagnosed, 75 gm of 2‐h OGTT should be repeated at 24–28 weeks (wks) of gestation (or any time a patient has signs and symptoms of hyperglycemia)	Nutrition and exercise therapy: If OGTT 2 h result is 153–199 mg/dL Medical therapy: start if 1‐h postprandial values exceed 130–140 mg/dL or 2‐h postprandial values exceed 120 mg/dL or fasting glucose exceeds 92 mg/dL persistently over 2 weeks. Early U/S: for dating and to check for congenital anomaly. Serial US: every 4–6 weeks and initiate fetal surveillance with BPP starting from 32 wks of gestational age. Delivery: Between 39 and 40 wks, but not later than 40 weeks. Referral: based on the reasons refer to in the guideline [17].

WHO guideline	Gestational DM: is diagnosed at any time in pregnancy if one or more of the following criteria are met. Fasting plasma glucose: 92–125 mg/dL (5.1–6.9 mmol/L). 1‐h plasma glucose: 180 mg/dL (10 mmol/L). 2‐h plasma glucose: 153–199 mg/dL (8.5–11.0 mmol/L).	Management: Nutritional therapy with exercise therapy and Insulin Therapy/Metformin as needed [13].

FIGO guideline	All women: should be screened for GDM by fasting plasma glucose, random blood glucose, or HBA1c at booking/first trimester. If it turns to be negative, perform 75 g of 2‐h OGTT between 24 and 28 weeks of gestation.	Lifestyle modification • If lifestyle modification fails, metformin, glyburide, or insulin should be considered as safe and effective treatment options for GDM. Insulin treatment: • The following insulin may be considered safe and effective treatment during pregnancy: Regular insulin, NPH, lispro, aspart, and detemir [14].

### 3.5. Healthcare Providers’ Awareness About GDM

The obstetricians and general practitioners were familiar regarding GDM’s etiology, symptoms, and management. They were also aware of the national guidelines and the procedures for managing and screening GDM.

“At first ANC contact, all pregnant women are assessed for risk of GDM, if a woman has risk factors for GDM; we perform screening using random blood glucose as soon as possible. If GDM is not diagnosed, we repeated the screening at their 26 weeks of gestation. The national guideline recommended the one step screening of GDM by using 75 g of oral glucose (OGTT), however it is not practiced in our hospital” (IDI#8, Obstetrician).

Why you have not put the national guideline into practice doctor?

“We know the presence of national guideline on GDM screening and management, and some healthcare professionals got the orientations on the management protocol. But it is not practiced by health professionals; negligence on the part of the healthcare provider to practice national guideline is among the main reasons for this (IDI#8, Obstetrician).”

According to the general practitioners and obstetricians, they stay up to date on the most recent advancements; in particular, national guidelines are updated on a regular basis through programs for continued medical education. They did not believe that additional instruction on GDM management and screening was necessary. However, middle‐level health professionals (midwives) felt that there was a shortage of relevant training, and they indicated a desire to resume formal GDM training tailored to the midwives’ position.

## 4. Methods of Education About GDM

All participants acknowledged that they do not have much time to teach pregnant women and their families about GDM. Nonetheless, they provided several strategies for increasing awareness of GDM. These included mass media (television and newspapers), educational films shown within the hospital, posters in the waiting area, and specialized counselors to teach patients about GDM. The most highly recommended course of action during antenatal care (ANC) days was to watch educational films regarding GDM. This approach excited the medical staff since it would provide women the opportunity to watch easily accessible and comprehensible films as they waited in the clinics. Additionally, it was thought that showing the movie on hospital property would make its messages more credible.

“It is good if all (Posters, flyers or video/ film etc.) would be available, as you know there is a high case‐flow in our hospital, and there is a lot of work load, all pregnant women who comes to this hospital seat at least 30 minutes in the waiting area, so I think it would be better if they are educate in the form of video/film in the waiting area” (IDI#6, general practitioner, age 38).

### 4.1. Barriers to GDM Screening and Managements

This study examines obstacles to early detection and treatment of GDM among healthcare providers. Barriers to the early diagnosis and treatment of GDM at various stages have been arranged using a socioecological model.

#### 4.1.1. Individual‐Level Barriers (Patient‐Related)

The study participants noted that women frequently do not perceive themselves as at risk for GDM and are unaware of its consequences, reducing their motivation to seek timely screening or adhere to management plans. They often initiate ANC late (third trimester) or receive fragmented care seeking ANC at local health centers or private clinics for convenience but delivering at tertiary hospitals. This fragmentation causes missed screening opportunities, as lower‐level facilities often lack GDM screening capacity. Furthermore, participants noted that contextual factors, the civile ware between the national army and FANO, have exacerbated delays in seeking ANC, directly impacting timely detection.

“Sometimes mothers show symptoms when they come to the hospital for childbirth as some women come to the hospital just for delivery service and follow their antenatal visit at nearby health center or private clinic. Private clinics don′t screen for GDM, just do a lot of ultrasound scans” (IDI#5, senior midwife, age 35).

Multiparous women, in particular, prioritize family duties over their health, choosing for quicker, local ANC visits that may not include GDM screening. The participating midwife mentions this discreet process:

“Now a day, many of mothers goes to health centers or private clinics to ANC follow‐up, and this challenge the early screening of GDM. Also, the lab is very busy (in hospitals), so the mothers may leave it in the middle” (IDI #4, midwife, 32 years old).

#### 4.1.2. Community/Cultural‐Level Barriers

GDM is often not viewed as a biomedical condition. Some attribute it to supernatural causes (e.g., Satan) or believe it is not serious during pregnancy, leading to denial of diagnosis and preference for traditional remedies (e.g., holy water). These cultural beliefs result in women not requesting screening and being reluctant to accept or adhere to medical treatment, placing the entire responsibility on providers to identify and encourage them.

#### 4.1.3. Health System–Level Barriers

**FIGURE 1:**
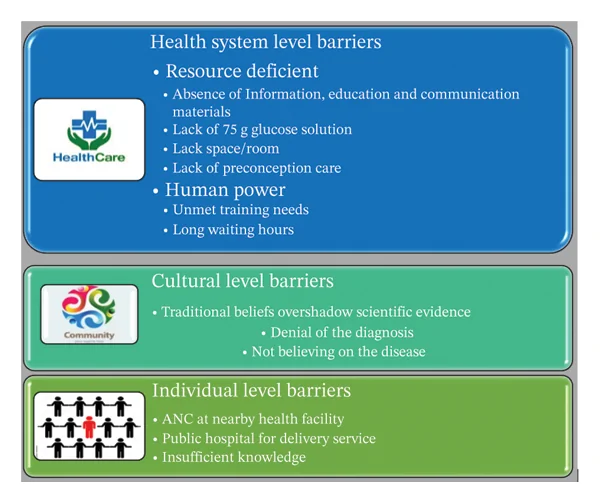
Thematic analysis of barriers to timely diagnosis of GDM based on the socioecological model. This figure is an original creation by the manuscript’s authors.

The majority of respondents reported that barriers to early detection and treatment GDM were lack of preconception care, lack of supply and equipment, lack of isolated screening area, unmet training needs, and shortage of trained healthcare providers. Figure 1 provides the summary of a thematic breakdown of obstacles based on socioecological model to GDM screening from the viewpoint of the healthcare providers.

### 4.2. Barriers for Management of GDM

The health system–related obstacles to managing GDM were shortage of trained healthcare providers, the unmet training needs of healthcare providers, shortage of drugs and equipment, insufficient knowledge skill of healthcare providers, lack of sustainable health education for mothers, high caseload that leads to long waiting time, and inadequate infrastructure, including the lack of space/room.

**FIGURE 2:**
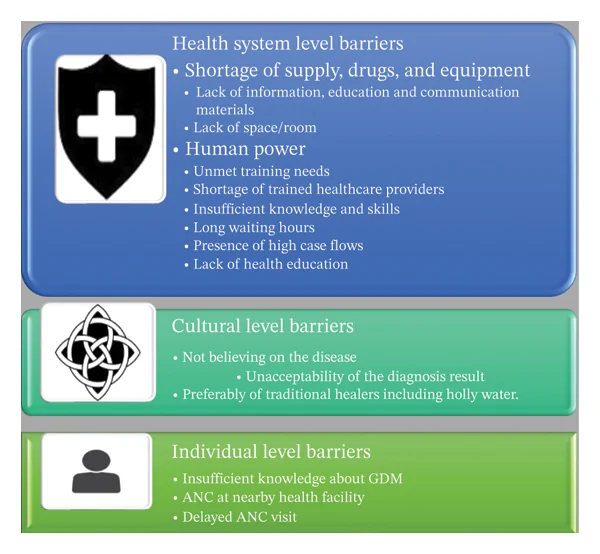
Thematic analysis of obstacles to gestational diabetes mellitus management utilizing the socioecological model. This figure is an original creation by the manuscript’s authors.

Individual‐level (patient‐related) challenges included delayed ANC visits, insufficient knowledge of GDM, and nonacceptance of the diagnosis. The cultural barrier was the concurrent use of traditional remedies, such as the sacred water of the sacred saint. In general, the problems with screening and managing the GDMs are manifold. The thematic analysis of barriers to early treatment of GBM using the socioecological model from the perspective of healthcare providers is presented in Figure 2.

Participants highlighted the obstacles and challenges of screening and management of GDM. One participant explained the issues as follows:

“I think gestational diabetes mellitus is not a major public health issue, the screening is not practiced by health professionals; because they did not understand the severity of the disease that can be caused by GDM. Another reason is that mothers are not asking for the screening because they are unaware of the problem. The lack of trained healthcare professionals is also a major concern in the area of early detection and treatment of GDM. In general, the presence of a high case flow in the hospital and negligence on the part of the healthcare provider to carry out timely screening are among the main reasons for this” (IDI#7, Obstetrician, age 42).

All study participants identified the problem of unmet training needs for healthcare professionals as an obstacle to the provision of comprehensive and timely health services, in particular for early detection and treatment of GDM. On‐job training is not accessible in hospitals; early guidance to healthcare providers on existing literature and management protocols is helpful for improving maternal health services as a whole. Explanations by one of the participants are given below:

“Timely orientation and sustainability of the job‐related training related to GDM is necessary, as it is necessary to support the healthy lifestyle of our clients. Not only that, but also the provision of training is mandatory for the provision of proper healthcare for women with GDM.” (IDI#, 38 years of age general practitioner)

Shortages of supplies, drugs, and equipment were also described as obstacles to GDM timely management. Lack of supplies and poor infrastructure, including lack of space or room, and glucose solutions were identified as problems in DM treatment during pregnancy. The lack of medicines in public hospitals can affect adherence, as most women cannot afford to buy medicines from private pharmacies because they are expensive.

“In our hospital, there is an unfriendly infrastructure, such as inaccessible room for screening GDM and/or the absence of exclusive diabetes ward. There may also be shortages of certain medicines. Although the national guidelines recommend a single‐step 75‐g oral glucose tolerance test (OGTT), we don’t have a 75 g glucose solution at our hospital” (IDI#1, midwife, 32 years of age).

Healthcare professionals have reported that most pregnant women are unaware of the screening process and the impact of GDM on their future health. As stated by the participants, most mothers do not believe in the disease, most women define diabetes mellitus as a disease caused by evil and can be treated with holy water and other traditional remedies, and therefore they (women) are often reluctant to take the medicines correctly. Women are often unaware of the risk of GDM during pregnancy and do not request an early screening. The following topic was discussed:

“Although, there may be shortages of medication, yet some mothers say, I won′t take it (the medication), I′m okay, I was okay; but they start with good advice and a deep relationship with the healthcare providers; first, a good relationship must be established, otherwise they will take the medicine the pharmacy store but, they won′t be able to use it. There is a problem of knowledge” (IDI #3, senior midwife, age 29).

We found that shortage of trained healthcare providers was a main barrier for proper management of GDM. The healthcare provider does not take full client history during their prenatal visits, which can be missed in order to screen for high‐risk mothers. A 6‐year‐old experienced participant also added the following:

“There is shortage of trained healthcare providers, which reduces the dissemination of information on GDM to pregnant women in advance. Some mid‐level providers are not even capable of providing counseling about self‐administering insulin.” (IDI #4, Midwife, 34 years).

In this study, late booking for ANC visit was identified as a major problem for early detection and treatment of GDM. Participants noted that some women have attended ANC care in nearby health centers or private clinics, and that the likelihood of early detection and treatment of GDM is lower in these settings.

“Sometimes mothers show symptoms when they come to hospital for giving birth, this is because some women come to hospital late or just for delivery service, and they follow their ANC service at the nearest health center or private clinic” (A 35 years old senior midwife).

## 5. Discussion

The rising global prevalence of GDM presents a terrible challenge to maternal and neonatal health, particularly in low‐ and middle‐income settings where resources are constrained [20, 21]. This study aims to explore healthcare providers’ perspectives on barriers to the early diagnosis and management of GDM in northwest Ethiopia.

The findings of this qualitative study reveal discordance between theoretical medical knowledge and clinical implementation regarding GDM in Northwest Ethiopia. While healthcare providers articulate a clear understanding of GDM‐related morbidities such as fetal macrosomia and long‐term maternal type‐2 diabetes, clinical practice remains tied to a reactive, crisis‐management model rather than the proactive screening encouraged by international standards. A critical tension exists between the Ethiopian National Guidelines and actual facility‐level implementation. Although the national protocol recommends a one‐step 75‐g OGTT [17], for high‐risk women, practice has devolved into selective screening using random blood sugar. This reliance on random blood sugar is clinically significant; it lacks the diagnostic sensitivity of the “gold standard” OGTT endorsed by WHO and FIGO [13, 14]; this may miss asymptomatic cases that do not present with overt hyperglycemic signs.

Significantly, only Debre Markos Comprehensive Specialized Hospital reported practicing universal screening. This suggests that institutional capacity and specialized designation exert a greater influence on guideline adherence than national policy itself. The result is a fragmented diagnostic landscape where a patient’s access to care is determined by a facility’s tier rather than her actual clinical risk. Ultimately, while the development of GDM protocols is a foundational achievement, bridging the implementation gap is the only way to mitigate adverse maternal and neonatal outcomes [32].

The current study found a cascade of barriers spanning individual, community and health system levels for early screenings of GDM. The most prominent patient‐level barrier for early screenings of GDM was a profound lack of awareness regarding GDM, its associated risks, and the importance of timely ANC. This knowledge deficit contributed to delayed ANC attendance, care‐seeking at facilities without diabetes screening capacity, and, in some cases, outright rejection of the diagnosis directly resulting in missed opportunities for screening. These findings align with previous research demonstrating that patient‐related factors substantially contribute to delayed GDM diagnosis [33]. Evidence from other studies also supports the barriers identified by our healthcare providers, including the absence of standardized guidelines and protocols, limited awareness of GDM among mid‐level providers, insufficient trained personnel, shortages of essential supplies and equipment, and late initiation of ANC [32, 34]. When women lack understanding of their risk, they are unable to participate meaningfully in shared decision‐making or proactively request screening, thereby shifting the entire burden of responsibility onto already overextended healthcare providers.

The finding revealed that mothers’ knowledge gaps are compounded by powerful community‐level cultural beliefs. In this foundation, GDM is frequently minimized as a nonserious condition or attributed to supernatural causes (e.g., Satan). This deeply held belief overshadows biomedical awareness, leading women to perceive no risk and thus not seek screening and timely managements. This finding supports evidence on how cultural perceptions hinder preventive care [35]. This underscores that health education must be culturally congruent, engaging not only pregnant women but also families and community leaders to shift normative beliefs [36].

In this study, the most frequently cited barriers for early detection and management of GDM was located at the health system level. These included shortages of trained professionals, unmet training needs, lack of essential supplies such as glucometers and test strips, and the absence of preconception care services. These findings are consistent with prior studies conducted in southern Ethiopia and India [37, 38]. System‐level shortages (e.g., lack of test strips) not only justify and reinforce provider reliance on selective screening but also contribute to community mistrust in the health system’s capacity to deliver quality care [39]. Moreover, the absence of targeted training for providers on updated GDM guidelines reflects a fundamental gap in health system stewardship, ensuring that available resources are not utilized optimally [40].

Healthcare providers generally prefer dietary modification and exercise as the primary approach to managing GDM, reserving medical treatment (insulin therapy) for cases where blood glucose levels cannot be adequately controlled through lifestyle interventions. This practice is consistent with international guidelines [14, 41, 42]. However, in the Ethiopian context, such a “preference” may also represent a pragmatic adaptation to systemic constraints, including limited capacity for frequent glucose monitoring, poor access to insulin, and lack of provider confidence in administering insulin therapy. These findings underscore that effective management of GDM requires more than awareness of clinical guidelines; it also demands tough system‐level support to ensure safe and consistent implementation.

### 5.1. Strengths and Limitations

This study has several notable strengths. First, it employed a multilevel analytical approach that examined barriers at the individual, interpersonal, community, and health system levels, allowing for a comprehensive understanding of the complex factors influencing GDM screening and management. Second, the inclusion of healthcare providers from primary, secondary, and tertiary facilities enabled the identification of important inconsistencies in screening practices across tiers of the health system insights that would not have emerged from single‐site studies. Third, experience‐based narratives from frontline healthcare providers generated nuanced explanations of how shortages of supplies, gaps in training, and inconsistent guideline dissemination shape real‐world practice. Finally, the study’s interpretation is deeply grounded in the Ethiopian health system context, enhancing the relevance of the findings for policymakers and program implementers working in similar resource‐constrained settings.

This study also has limitations that should be considered when interpreting the findings. Because data were collected from selected facilities within one region, the results may not fully represent the diversity of practices and challenges across all regions of Ethiopia. The reliance on provider perspectives may introduce social desirability bias, as participants might underreport their own knowledge gaps or overemphasize system‐level constraints. Additionally, although the study identifies patient‐ and community‐level barriers, these insights are based on provider accounts rather than direct interviews with pregnant women or community members, which may limit the depth of understanding regarding cultural beliefs and care‐seeking behaviors.

## 6. Conclusion

Early detection and management of GDM in Northwest Ethiopia remain constrained by a complex interplay of individual knowledge gaps, cultural norms, and systemic health service weaknesses. Addressing these challenges requires a comprehensive health system approach that concurrently strengthens infrastructure, expands human resource capacity, and fosters community engagement. Such an integrated strategy is essential to translate national policy into equitable and effective practice, ultimately improving maternal and neonatal outcomes.

**Nomenclature**
ANC: Antenatal care
DM: Diabetes mellitus
FIGO: International Federation of Gynecology and Obstetrics
GDM: Gestational diabetes mellitus
IDI: In‐depth interview
WHO: World Health Organization

## Author Contributions

Getachew Tilaye Mihiret: conceptualization, designed the study, methodology, software, formal analysis and interpretation of the data, validation, writing–original draft, and writing–review and editing. Kumlachew Solomon Wondmu: designed the study, methodology, formal analysis and interpretation of the data, writing–original draft, and writing–review and editing. Fekadu Baye: designed the study, methodology, and writing–review and editing. Mulunesh Minale: designed the study, methodology, formal analysis and interpretation of the data, and writing–review and editing. Mastewal Yechale: designed the study, methodology and interpretation of the data, and writing–review and editing. Misganaw Fikirie Melese: designed the study, methodology, and writing–review and editing. Yilkal Dagnaw Melesse: designed the study, methodology, and writing–review and editing. Aysheshim Belaineh Haimanot: designed the study, methodology, and writing–review and editing. Aderajew Agmass Adebabay: designed the study, methodology, and writing–review and editing. Haile Amha: designed the study, methodology, and writing–review and editing.

## Funding

Debre Markos University was funded only for personnel costs.

## Disclosure

The lead author (GTM) affirms that this manuscript is an honest, accurate, and transparent account of the study being reported; that no important aspects of the study have been omitted; and that any discrepancies from the study as planned. All authors have read and approved the final version to be published, and they have agreed on the journal to which the article has been submitted. The funders had no role in the study.

## Consent

The authors have nothing to report.

## Conflicts of Interest

The authors declare no conflicts of interest.

## Supporting Information

Additional supporting information can be found online in the Supporting Information section.

## Supporting information


**Supporting Information** Two supporting files accompany this manuscript. Supporting File‐1 contains the interview guide used for the in‐depth interviews with healthcare providers. Supporting File‐2 presents the coding framework developed during the thematic analysis, including the coding structure and theme development process.

## Data Availability

The data supporting the findings of this study are available within the article and in the supporting information. Due to the qualitative nature of the data and confidentiality agreements, interview transcripts are not publicly available.
