# Supplementary material for: Barriers to Early Detection and Management of Gestational Diabetes Mellitus in Northwest Ethiopia: A Qualitative Study From the Healthcare Providers’ Perspectives
Source: Obstet Gynecol Int. 2026 Aug 27;2026:1739878. doi: 10.1155/ogi/1739878 (PMC13519957; doi:10.1155/ogi/1739878)
Supplement: Supplementary file 1 [file OGI-2026-1739878-s001.zip › Supplementary File 2_Coding framework.docx]

# Supplementary ****File 2****: Coding framework used during thematic analysis, including initial codes, axial categories, and illustrative quotes aligned with grounded theory methodology.

# Evolved Grounded Theory Coding Framework for GDM Screening and Management

**Context:** Healthcare Providers' Perspectives on the Early Detection and Management of Gestational Diabetes Mellitus in Northwest Ethiopia.

# Open Coding: derived through line-by-line analysis of interview transcripts.

| Code | Definition | *Representative Quote* |
| --- | --- | --- |
| Fearing blame | Hesitation to disclose or act due to fear of consequences | *“If something goes wrong, we’re blamed.”* |
| Sending to lab | Referring clients to laboratory for GDM screening | *“We send them to the lab when symptoms appear.”* |
| Lack of IEC materials | Absence of educational tools specific to GDM | *“We don’t have IEC materials for GDM.”* |
| Disbelief in diagnosis | Mothers reject or delay acceptance of GDM diagnosis | *“Most mothers don’t believe the diagnosis.”* |
| Adjusting diet | Non-pharmacological treatment strategy | *“They prefer to change their diet rather than take insulin.”* |
| No fixed screening schedule | Screening occurs, not routinely | *“We do not have a fixed day for GDM screening.”* |
| Lack of training | No formal GDM-related instruction for midwives | *“We haven’t received any training on GDM.”* |
| Referral to medical ward | Midwives link clients to physicians for follow-up | *“We just refer them to the medical ward.”* |
| Symptom-based screening | Screening triggered only by visible symptoms | *“If she shows symptoms, we send her to the lab.”* |
| Urban susceptibility | Perception that urban mothers are more vulnerable | *“We mostly see GDM in urban women.”* |
| Long waiting times | Delays in lab or treatment due to system overload | *“Sometimes they wait too long for lab results.”* |
| Lack of provider knowledge | Midwives unaware of GDM protocols or guidelines | *“We don’t know the national guidelines.”* |

# Axial Coding

**Categories developed by clustering related open codes using the paradigm model**.

| Axial Category | Related Codes | Causal Conditions | Contextual Factors | Consequences |
| --- | --- | --- | --- | --- |
| Screening Practices | - Sending to lab, - Symptom-based screening, - No fixed schedule | Lack of routine protocols | Overburdened ANC workflows | Missed early detection |
| Provider Roles & Limitations | - Referral to medical ward, - Lack of training, - Lack of knowledge | Hierarchical care norms | Absence of role clarity | Fragmented care delivery |
| Client Experience & Barriers | - Disbelief in diagnosis, - long waiting times, - Urban susceptibility | Low health literacy | Cultural beliefs, geographic disparities | Delayed treatment initiation |
| Treatment & Management | - Adjusting diet, - Insulin reluctance, - Follow-up challenges | Fear of medication, stigma | Limited counseling tools | Suboptimal glycemic control |
| Health System Gaps | - Lack of IEC materials, - Lack of training, - No guideline awareness | Weak policy implementation | Resource constraints | Inconsistent GDM care quality |

# Sample Analytic Memos

| Memo Title | Content |
| --- | --- |
| Memo 1: Role Deferral | Midwives consistently defer GDM education to physicians, suggesting entrenched role boundaries and lack of empowerment. |
| Memo 2: Reactive Screening | Screening is symptom-driven, not protocol-based indicating a reactive care model shaped by resource constraints. |
| Memo 3: Cultural Resistance | Client disbelief in diagnosis reflects deeper cultural narratives around pregnancy and illness, requiring tailored education. |
| Memo 4: Systemic Fragmentation | The absence of IEC tools, training, and guideline awareness points to a fragmented health system that fails to support midwives. |
